# Presentation and management of traumatic breast hematomas: a case series and review of the literature

**DOI:** 10.1093/jscr/rjag961

**Published:** 2026-10-27

**Authors:** Amanda Rose Camarda, Vincent Marcucci, Taylor Ouellette, Alexandra Shapiro, Kathryn Eckert, Roshani Patel

**Affiliations:** Department of Surgery, St. George’s University School of Medicine, Grenada, West Indies; Department of Surgery, Jersey Shore University Medical Center, Neptune, NJ 07753 United States; Department of Surgery, Jersey Shore University Medical Center, Neptune, NJ 07753 United States; Department of Surgery, Jersey Shore University Medical Center, Neptune, NJ 07753 United States; Department of Breast Surgery, Jersey Shore University Medical Center, Neptune, NJ 07753 United States; Department of Breast Surgery, Jersey Shore University Medical Center, Neptune, NJ 07753 United States

**Keywords:** blunt breast trauma, penetrating breast trauma, traumatic breast hematoma, internal mammary artery

## Abstract

Breast hematomas resulting from traumatic injuries are poorly characterized and variably managed. Traumatic hematomas are less common than iatrogenic hematomas and lack a clear algorithm for assessment and management. Hematoma management in trauma scenarios is primarily guided by concern for ongoing blood loss and resultant variation in vital signs. As a result, management decisions are less likely to incorporate concerns about scarring and cosmesis, though these are commonly critical parts of breast care. Through a series of patients evaluated at a level one trauma center, this article explores the appropriate assessment, management, and follow-up for patients with breast trauma and resultant hematomas. We propose several strategies to address care of immediate traumatic injuries while also optimizing long-term breast health.

## Introduction

There is a paucity of data available regarding the optimal management of traumatic breast hematomas (TBH). While treatment of most traumatic injuries is guided by data-driven algorithms, no such framework exists for TBH. As a result, these conditions remain underreported and are not well tracked [1].

In the surgical literature, iatrogenically caused breast hematomas, those resulting from procedures like core needle biopsy or surgical excision, occur in ~1% of all cases and are thought to be more common than those resulting from trauma [2, 3]. Overall, TBH comprise a smaller proportion of all breast hematomas. When TBH are found, it most commonly are the result of blunt trauma (i.e. motor vehicle accident resulting in seatbelt injury), though penetrating trauma may also occur [2, 4–8]. Several risk factors including advanced age, elevated body mass index, hypertension, tobacco use, concomitant malignancy, bleeding disorders, and anticoagulant therapy predispose patients to hematoma development in scenarios of both blunt and penetrating trauma [9].

Regardless of the mechanism of traumatic injury, TBH occur as a result of damage to the arterial or venous blood supply to the breast. The arterial blood supply is derived from the lateral thoracic branch of the axillary artery, branches of the internal mammary artery (IMA), the thoracromial artery, the thoracodorsal artery, and intercostal perforators [10]. There is a perception that the majority of TBH will undergo spontaneous resorption and resolve without intervention. While this may apply to smaller injuries, larger or actively expanding TBH may require additional intervention. In 2007, Majeski proposed a classification for TBH with four categories of injuries where a mild injury, such as a crush injury (Class I), could be managed with observation and supportive care while a severe injury, such as a transection (Class III) or avulsion (Class IV), would be managed with interventional radiology (IR) and/or surgical intervention [11]. While this classification supports the role of operative management in dramatic cases that include open wounds or active bleeding, it does not capture subtle differences based on injury size and symptoms, which may also significantly impact outcomes. A delay in diagnosis and definitive management of TBH can lead to infection, skin necrosis, scarring, fat necrosis, and prolonged pain. This can have downstream consequences on breast imaging where non-malignant calcifications can develop, complicating subsequent breast cancer screening [4, 12]. TBH have also been associated with refractory hypotension and hemorrhage in female trauma patients, especially those on anticoagulation [13].

Current management strategies available for the treatment of TBH include conservative/expectant management (i.e. application of warm compresses, compression dressings, blood transfusions), surgical management (i.e. washout with hematoma evacuation), or IR management (i.e. angioembolization) [1, 4, 6, 11, 14].

## Case presentations

### Case 1

A 55-year-old female, with a history of non-small cell lung cancer with adjuvant chemotherapy, tobacco use, myocardial infarction, and home oxygen use, presented after a high speed motor vehicle collision (MVC) where she was the restrained front seat passenger. She was not on anticoagulation. On examination, she was noted to have a seat belt sign and mild confusion. She was not found to have any significant traumatic injuries and was discharged. Incidentally, her trauma work-up revealed progression of her lung cancer with imaging findings suspicious for distant metastatic disease in the brain and adrenal glands. She was referred for outpatient breast surgery follow-up due to concern for axillary adenopathy and breast mass in the setting of lung cancer progression. Physical examination revealed circumferential left breast ecchymosis and a 5 cm hematoma consistent with TBH due to blunt trauma (Fig. 1A and B). Left axillary adenopathy was not appreciated on clinical exam. Due to her comorbidities, a minimally invasive approach to drainage was elected with IR drain placement (Fig. 1C) and antibiotic prophylaxis. The drain remained in place for 10 days with near resolution of the hematoma cavity on follow-up imaging and significant improvement in her corresponding clinical exam (Fig. 1D). Unfortunately, the patient passed shortly after drain removal due to sequelae from metastatic lung cancer, therefore limiting additional follow-up.

**Figure 1:**
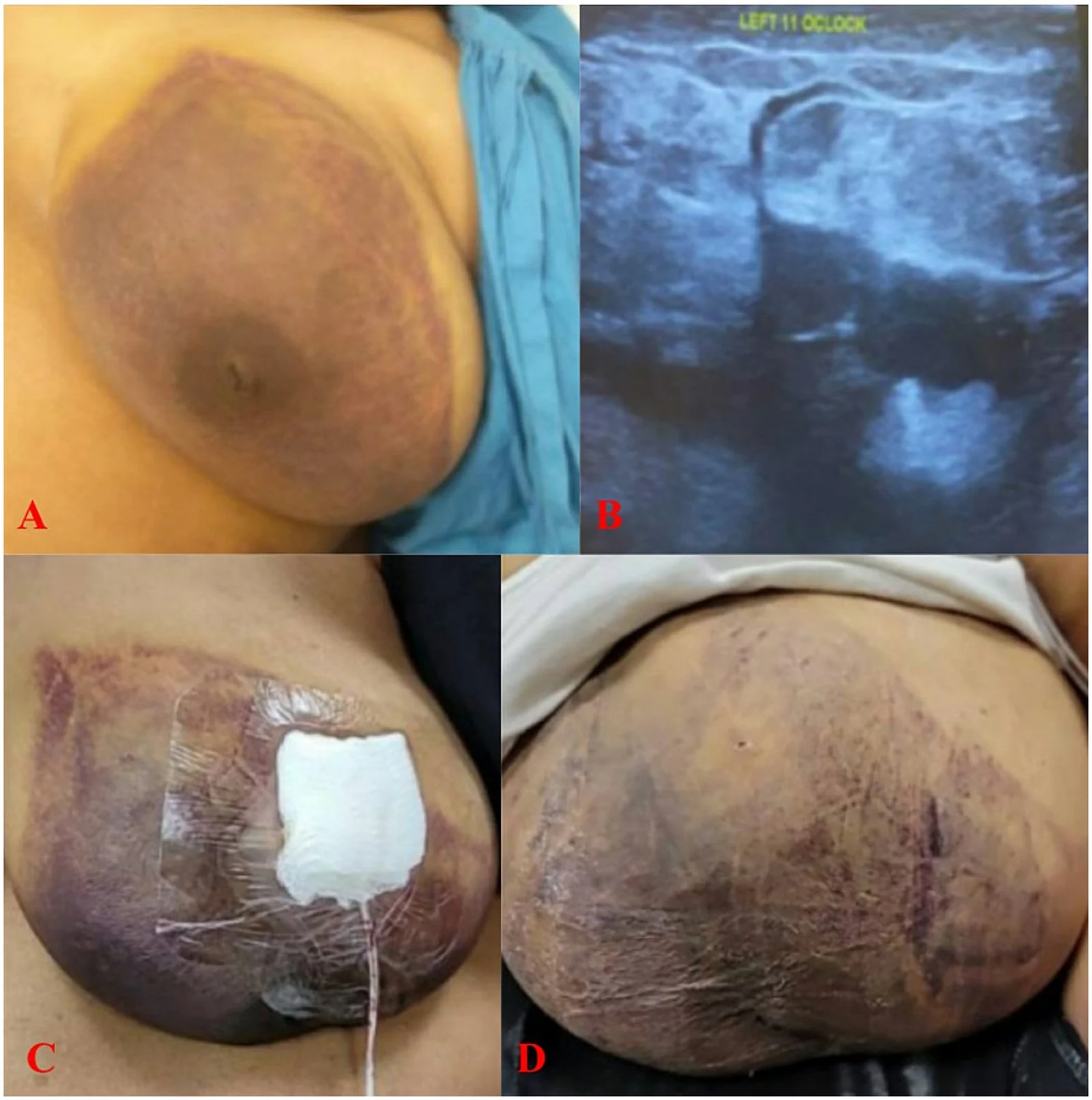
(A) Clinical photo at initial presentation to outpatient breast surgery office several weeks after MVA; (B) corresponding ultrasound performed at time of presentation; (C) clinical photo after IR drain placement; (D) clinical photo after drain removal demonstrating significant improvement in ecchymosis.

### Case 2

A 63-year-old female, without history of anticoagulation use, presented after an MVC where she was the restrained driver. She complained of left breast pain, cervical/thoracic pain, and dizziness upon arrival. Computed tomography angiography (CTA) completed as part of her trauma work-up, demonstrated an expanding right breast hematoma with active extravasation of blood into the hematoma (Fig. 2A). The patient was initially managed with non-operative interventions by the trauma team including serial hemoglobin monitoring and a compressive dressing. IR evaluated the patient and determined embolization was not feasible due to the vessel’s distal nature. The patient did not require blood transfusion and remained hemodynamically stable throughout her admission. She was seen by the trauma service for outpatient follow-up 2 weeks later where concern for a large, symptomatic breast hematoma persisted. Subsequent evaluation by breast surgery was remarkable for a large residual hematoma with signs of impending skin breakdown (Fig. 2B). Ensuing operative drainage resulted in 500 cc of hematoma evacuated. Extensive debridement of necrotic skin was completed at the same time. The patient progressed well postoperatively and ultimately had resolution of both the overlying skin findings and hematoma cavity 46 days following her initial injury (Fig. 2C).

**Figure 2:**
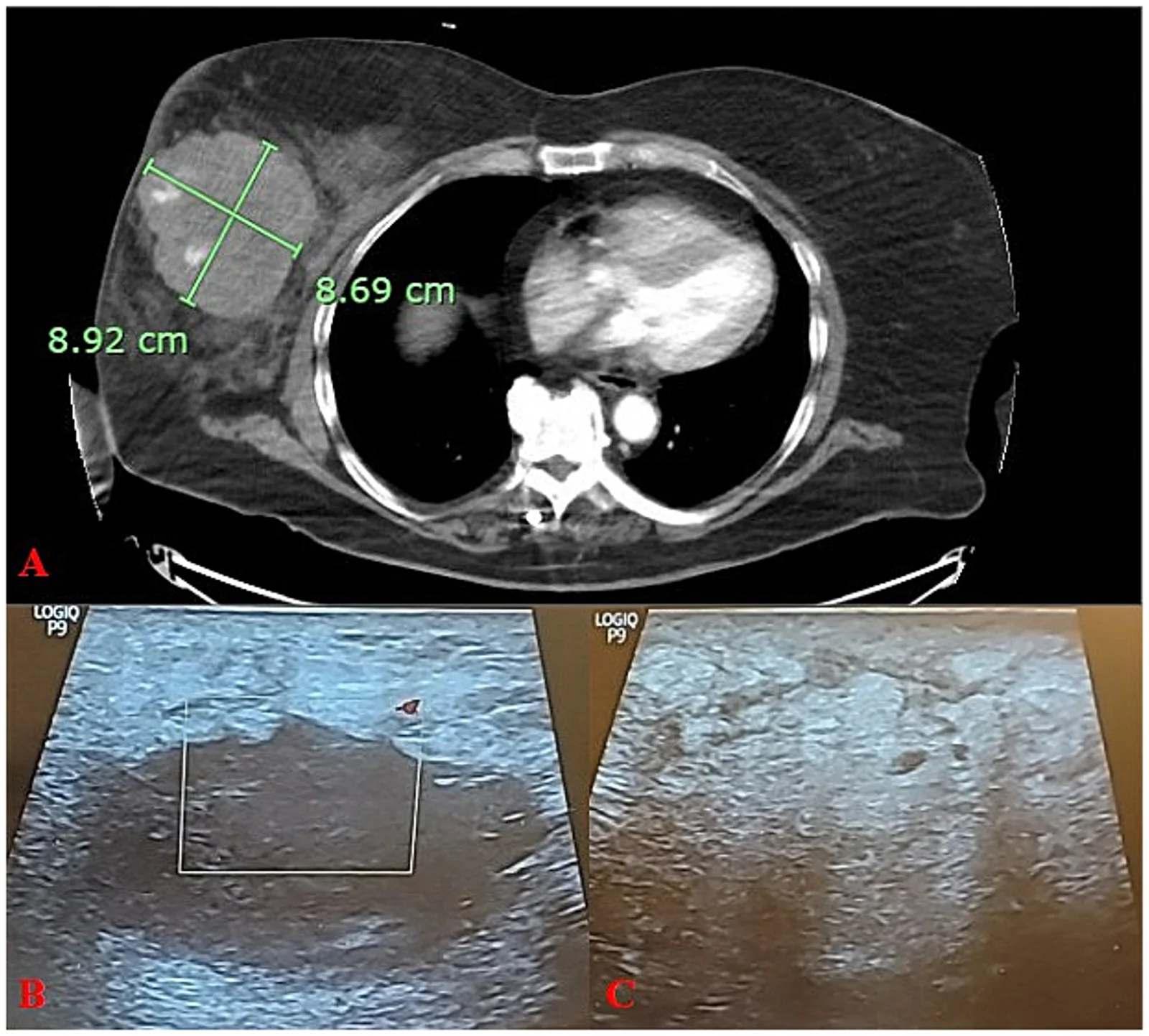
(A) CTA at presentation demonstrating large hematoma with active extravasation; (B) point of care ultrasound demonstrating large residual hematoma at time of outpatient breast surgery evaluation >2 weeks after initial injury; (C) resolution of hematoma cavity noted on follow-up postoperative ultrasound.

### Case 3

A 78-year-old female, with no significant medical history, presented after slipping and sustaining blunt trauma to her left chest/flank. CTA of the chest showed a mass at the inferior aspect of the left breast, measuring 1.7 × 2.2 cm (Fig. 3A). No other traumatic injuries were appreciated and the patient was discharged. She was referred to breast surgery for evaluation to rule out neoplastic process versus traumatic etiology of the radiographic breast finding. She underwent mammography and a left breast ultrasound, which demonstrated a 1.5 cm cystic lesion, consistent with a resolving hematoma at the site of blunt trauma and resultant skin ecchymosis (Fig. 3B). She improved with non-operative management including compression and cold compresses. She was followed clinically and experienced complete resolution of her left TBH.

**Figure 3:**
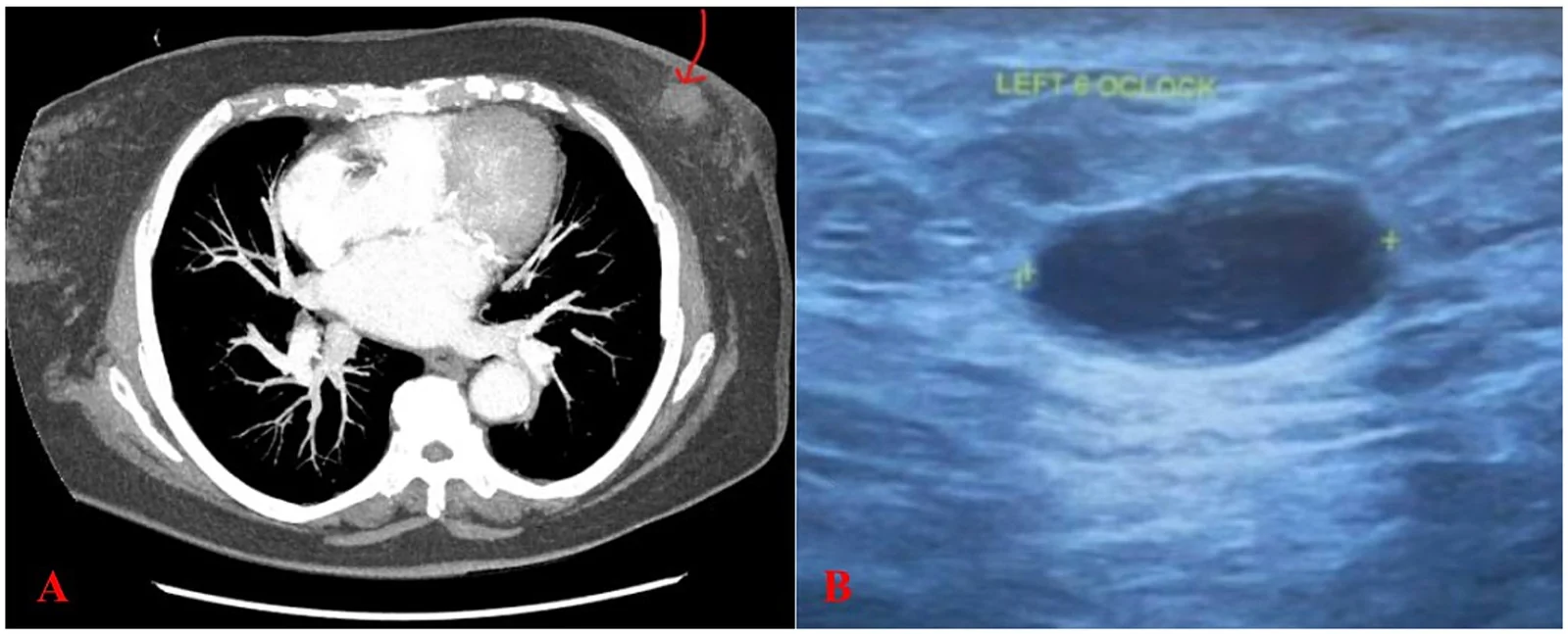
(A) CTA on initial presentation demonstrating 1.7 × 2.2 cm breast mass; (B) follow-up outpatient ultrasound 1 month later demonstrating small cystic lesion measuring 1.5 cm corresponding to CTA finding.

### Case 4

A 71-year-old female, with no pertinent medical history, presented for breast surgery evaluation 2 weeks after sustaining a horse bite to her right medial upper breast. On physical examination, she had significant right breast pain and right upper breast ecchymosis with underlying hematoma (Fig. 4A). She was taken for surgical evacuation due to pain and failure to resolve with non-operative management. Intra-operatively, 200 cc of organized hematoma was evacuated. SPY (real time imaging used to look at perfusion to tissue with the use of a dye and camera) angiography was performed and revealed a branch of the IMA had been transected (Fig. 4B). As a result, an area of necrotic skin was identified and excised. No active bleeding was noted on angiography. A drain was placed at the time of closure and removed in the office 1 week later at outpatient follow-up (Fig. 4C). The patient was noted to be healing well with significant improvement in pain postoperatively (Fig. 4D).

**Figure 4:**
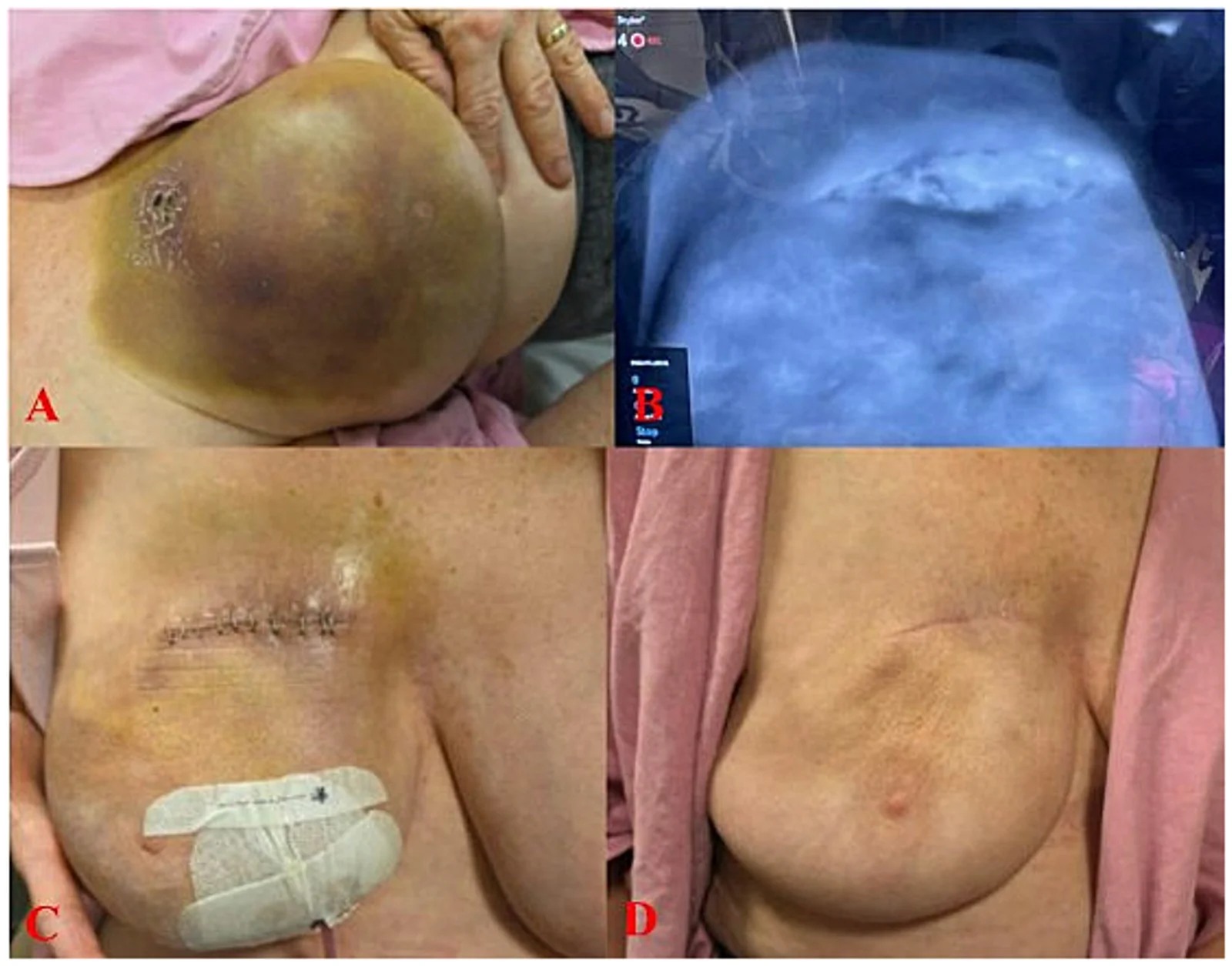
(A) Clinical photo at initial presentation 2 weeks after right breast horse bite injury; (B) intra-operative SPY angiography findings demonstrating internal mammary transection and resultant periincisional hypoperfusion; (C) clinical photo 10 days postoperative with surgical drain in place; (D) clinical photo at 1 month postoperative demonstrating resolution of hematoma with near complete resolution of corresponding skin ecchymosis and appropriately healing, well-perfused incision.

### Case 5

An 83-year-old female, with prior history of breast cancer (left breast lumpectomy, left axillary sentinel lymph node biopsy, and adjuvant radiation/endocrine therapy), atrial fibrillation (on apixaban), and hypertension, presented after MVC without airbag deployment. CTA completed for trauma evaluation demonstrated a grade 4 liver laceration, sternal fracture, peripancreatic edema, and a large left breast hematoma (Fig. 5A). Her apixaban was reversed with four-factor prothrombin complex concentrate and she received three units of packed red blood cells. Angiography demonstrated extravasation from the left IMA (Fig. 5B) in addition to the left gastroepiploic and left hepatic arteries. Coil embolization was successfully performed by IR for all three arteries and subsequent evaluations of hemoglobin level remained stable (Fig. 5C). Unfortunately, the patient remained symptomatic with significant left breast pain for several days after embolization and was subsequently evaluated by breast surgery. She was found to have a large residual hematoma encompassing ~70% of her left breast on examination. Surgical drainage was offered, but declined by the patient. As a result, percutaneous drainage by IR was pursued instead. A total of 150 cc of liquefied hematoma was drained prior to discharge. An additional 300 cc of output was noted during outpatient follow-up. Approximately 3 weeks after discharge, the catheter stopped functioning and was removed. A 100 cc of hematoma was manually expressed from the drain site at the time of removal. The patient noted persistent pain after the drain was removed and eventually agreed to surgical drainage of the retained hematoma. An additional 50 cc of hematoma was evacuated at the time of surgery and a surgical drain was placed. The surgical drain was removed in the outpatient setting 1 week postoperatively and the patient experienced significant clinical improvement.

**Figure 5:**
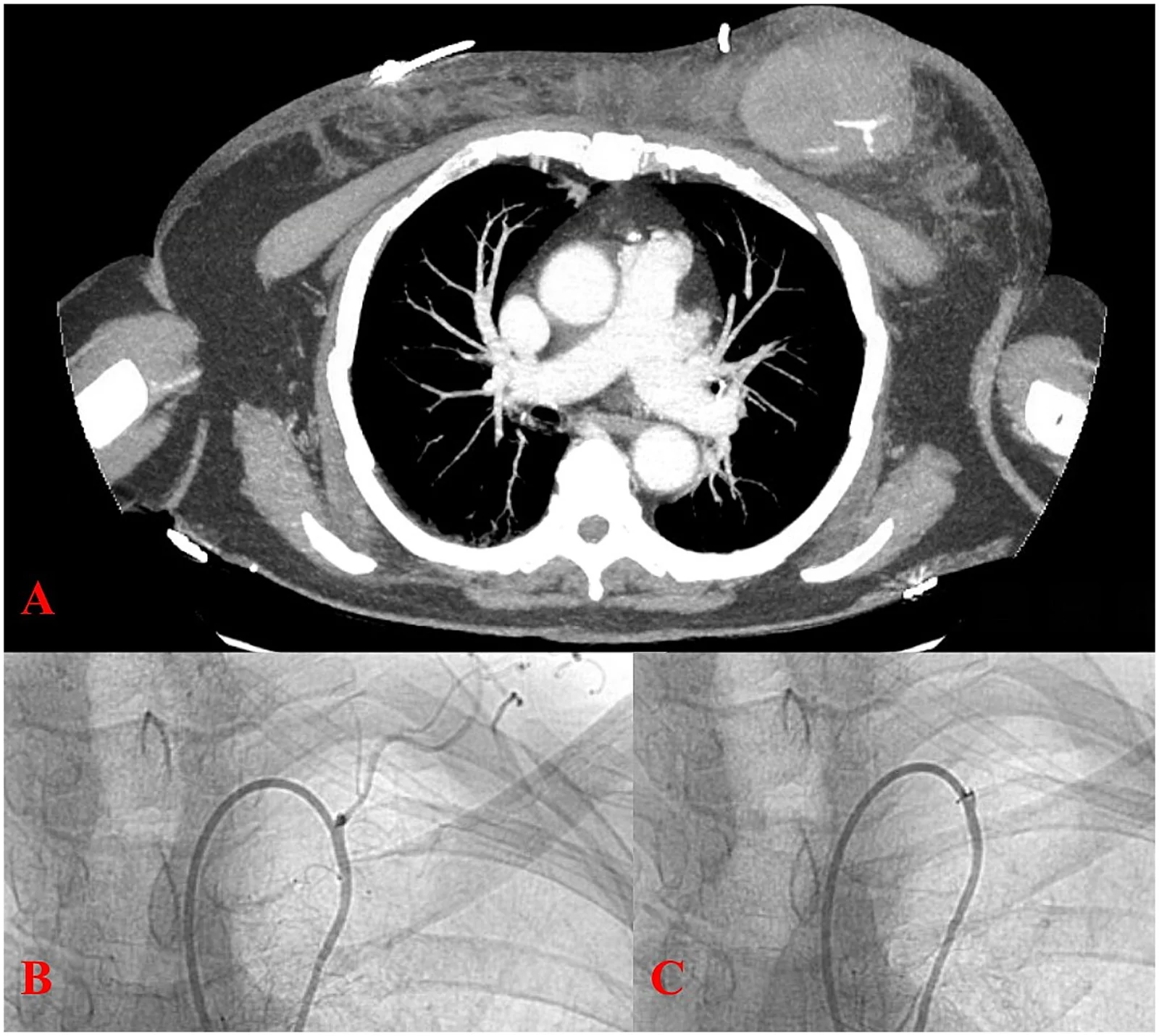
(A) CTA demonstrating active extravasation into left breast hematoma after MVC; (B) IR catheter directed angiography demonstrating extravacation from the left IMA; (C) IR catheter directed angiography postcoil embolization demonstrating no further extravasation.

## Discussion

Though TBH remains rare, the optimal management of TBH is controversial. As illustrated by this case series, the lack of clear guidelines regarding management of TBH often results in patients experiencing delays in definitive management. Patients with very large hematomas in this case series benefited from more aggressive interventions either with drain placement by IR or surgical washout.

Not all patients with clinically significant TBH will experience disturbance in vital signs, which is well illustrated in this case series [1, 6, 14]. In trauma, hypotension and tachycardia are typically inflection points in treatment which prompt more aggressive measures. Without reliable changes in these metrics to guide management, TBH patients often are managed without intervention ultimately resulting in delayed resolution. Leech *et al*. performed a 10-year retrospective study examining patients with iatrogenic, postsurgical hematomas. Large hematomas (those greater than 489 ml) did not necessarily present with hypotension or tachycardia, though were symptomatic by patient reporting. Some of these patients even experienced vasovagal responses secondary to significant pain [7].

While there is no absolute size threshold to differentiate hematoma size and intervention, much of the decision to proceed with interventional procedures is based on patient symptoms such as pain and swelling in addition to imaging findings. In studies of patients who have developed hematomas after vacuum-assisted core needle biopsies, there is a demonstrated relationship between the size of the postintervention hematoma and its appropriate management. Zheng *et al*. found that smaller hematomas (<2 cm) resolved in 1 month or less while larger hematomas (3–4 cm in size), could take up to 6 months to resolve [8]. Guzmán-Aroca *et al*. followed 56 patients with hematomas greater than 2.5 cm in size. Of these, 50 patients were observed and had resolution of hematoma by 6 months, while the remaining six patients were significantly symptomatic and required hematoma evacuation within 2 weeks [15].

These findings were echoed in our case series. In Case 3, the patient developed a small TBH (2.2 cm) which resolved within a short period of time with nonoperative interventions. The majority of patients included in this series (Cases 1, 2, 4, and 5) experienced larger hematomas that all required more aggressive intervention with either IR drain placement or surgical evacuation. Unfortunately, in spite of the large presenting size of hematomas in these patients, they were often managed initially with conservative interventions like compression alone. All of the patients included in this series experienced delays in definitive management of their hematomas, often in excess of 2 weeks after initial hematoma identification. This highlights the importance of prompt attention and management to avoid a protracted post-TBH course including pain, compromised skin viability, prolonged drain requirements, poor breast cosmesis, and long-term changes to the breast parenchyma.

In conclusion, we propose that early surgical drainage should be considered for patients with large hematomas (>3 cm in size), possibility of skin breakdown, significant/prolonged pain, poor cosmetic outcomes, and potential for significant scarring. While we cannot extrapolate generalizable data from this small case series, our experience and review of the literature suggest that if operative or IR drainage is considered at the time of the inciting trauma rather than taking an expectant approach, more optimal patient outcomes may be achieved. Further research and multidisciplinary collaboration are needed to establish assessment and treatment algorithms that may become the gold standard for management of TBH.
